# Posttraumatic Growth in Psychosis

**DOI:** 10.3389/fpsyt.2016.00202

**Published:** 2016-12-19

**Authors:** Yael Mazor, Marc Gelkopf, Kim T. Mueser, David Roe

**Affiliations:** ^1^Faculty of Social Welfare and Health Sciences, Department of Community Mental Health, University of Haifa, Haifa, Israel; ^2^Department of Occupational Therapy, Psychology, and Psychiatry, Boston University Center for Psychiatric Rehabilitation, Boston, MA, USA

**Keywords:** posttraumatic growth, psychosis, psychopathological symptoms, meaning making, coping self-efficacy

## Abstract

**Objective:**

Recent research has shown high rates of exposure to trauma among people with serious mental illness (SMI). In addition, studies suggest that psychosis and mental illness-related experiences can be extremely traumatic. While some individuals develop full blown PTSD related to these experiences, it has been noted that some may also experience posttraumatic growth (PTG). However, few studies have examined PTG as a possible outcome in people who have experienced psychosis.

**Method:**

To further understand the relationships between psychosis and PTG, 121 participants were recruited from community mental health rehabilitation centers and administered trauma and psychiatric questionnaires.

**Results:**

High levels of traumatic exposure were found in the sample. Regarding our main focus of study, we observed that people who endured psychosis can experience PTG, and that PTG is mediated by meaning making and coping self-efficacy (CSE) appraisal. Psychotic symptoms were found to be a major obstacle to meaning making, CSE, and PTG, whereas negative symptoms were found to be significantly related to PTG when mediated by meaning making and CSE.

**Conclusion:**

The current research provides preliminary evidence for potential role of meaning making and CSE as mediators of PTG in the clinical, highly traumatized population of people with SMI who have experienced psychosis. This may have both research as well as clinical practice relevance for the field of psychiatric rehabilitation.

## Introduction

Research has suggested that psychosis and mental illness-related experiences can be extremely traumatic (1–3). As in the case of traumatic life events in non-clinical populations (4), psychosis could theoretically also lead to posttraumatic growth (PTG). PTG consists of five facets of growth: appreciation of life, relating to others, new found possibilities in life (for example, a new career pursuit following trauma), personal strength, and spiritual change (5, 6). PTG occurs after a trauma, which may have significant negative impact, but at the same time be the starting point of an emotional and behavioral rebuilding process, through PTG (7). The promotion of PTG may contribute to the treatment of people who experienced massive traumas such as people with serious mental illness (SMI) who have endured psychotic symptoms. Although multiple studies have found that traumas are frequent and repetitive experiences in these individuals (8, 9), and qualitative studies on the experiences of recovery have reported elements of growth in this population (10, 11), only few have explored the possibility of PTG in people with SMI who have experienced psychosis.

Psychotic symptoms are a common feature of SMI, most prominent in schizophrenia, but frequently present in other disorders as well, such as bipolar disorder and major depression. Individuals who have experienced psychosis are also more likely to have been exposed to trauma (12) and are more vulnerable to developing posttraumatic symptomatology (8, 13–16). The impact of exposure to traumatic events has been found to worsen the characteristic symptoms of SMI, such as grandiose or paranoid delusions, hallucinations, anxiety, and depression and to contribute to general psychopathology (e.g., depression, anxiety, impaired social judgment, etc.) in psychiatric disorders such as cognitive deficits, risk taking behaviors, and the experience of psychosis itself (8, 17–20). A possible explanation to the adverse impact of traumatic exposure on SMI symptoms is the “social defeat hypothesis of Schizophrenia” (21). This approach suggests that long-term exposure to the experience of social defeat or exclusion can lead to the sensitization of the mesolimbic dopamine system, whereby exposure to a given stimulus (e.g., stressor), may result in an increased response to subsequent exposures. Continuous humiliation and seclusion, which are inherent in childhood traumas, such as abuse, can indeed result in feelings of social defeat, and therefore repeated traumatic exposure may be one of the reasons of the aggravation of SMI symptoms (12).

Psychopathological symptoms can appear in the form of both positive and negative symptoms. Positive (or psychotic) symptoms can be experienced as severely traumatic on account of reality distortions such as delusions and hallucinations (3, 22–25), and can contain adverse and frightening, and potentially traumatic content.

Negative symptoms could also contribute to the traumatic experiencing of psychosis. These symptoms manifest in a lack of energy, drive, and interest, which are caused by a basic inhibition in motivation (26–28). Amotivation is one of the negative symptom groups and is specifically relevant to the possible traumatic sequelae of psychosis. These include anhedonia, lack of social drive and social isolation, and treatment non-adherence (8, 29, 30). This chain of events might elevate the risk for relapse and indirectly lead to traumatic experiences that are part of psychosis (3, 8).

Alongside, the adverse consequences of psychopathological symptoms are the unique outcome of coping with adversity (31), namely, PTG. PTG manifests itself after trauma through one’s thought processes, emotions, and behaviors, and it may include both a constructive and an illusory aspect (32). PTG is facilitated by the reduction of emotional distress associated with traumatic experiences, which allows ruminations to transform into deliberate thinking about the trauma and its aftermath. As a result, people may feel that they have changed positively following their traumatic experience (31, 33). In addition, PTG can also express itself as concrete actions whereby people act in new and different ways in the face of trauma (34, 35). Although traumatic experiences are common in people who have experienced psychosis—studies have suggested that up to 98% of people who experienced psychosis were exposed to prior trauma (36), only a few studies investigated PTG in this population (37–39). Thus, we still lack the understanding of what might facilitate PTG in these individuals.

One important factor that may contribute to the development of PTG is coping self-efficacy (CSE), which has been found to be positively correlated with PTG (40). CSE is the perceived or appraised ability to manage one’s personal functioning and environmental demands following traumatic or stressful events, through cognitive processes and changes in one’s belief system (41, 42). These processes can provide a sense of control and actual control in the face of traumatic adversity, reducing vulnerability to being overwhelmed by helplessness (42, 43). CSE includes three dimensions: (a) self-appraisal of coping capabilities; (b) transformative actions [e.g., the ability to change or maintain one’s coping style when faced with different challenges (43)]; and (c) thought control efficacy (i.e., the sense of control people have over their thoughts, and their ability not to be influenced continuously by cognitive intrusions). Research suggests that the ability to accept situations that cannot be changed is a major aspect in adaptation to uncontrollable events, and that the reappraisal of coping in a more positive light is one path to PTG (32, 44). An important question is whether individuals who experience psychosis and therefore might suffer from stereotyped thinking, delusions, preoccupation, and lack of insight, might be hampered in their ability to develop self-appraisals, transformative actions, and the belief in one’s ability to control one’s thoughts (i.e., CSE), and therefore possibly PTG.

Another major factor that is both protective and facilitative of PTG is meaning making. The need to search for meaning is a basic human tendency (45), which may be activated after the violation of basic life assumptions regarding oneself and the world following trauma (46–49). Definitions and measures of meaning making vary throughout theories (50); however, all theoreticians agree that meaning is a crucial component in confronting adversity and promoting growth (51). The inability to find meaning in life after encountering adverse situations can result in extreme distress (45). Studies show that individuals with SMI are capable of searching for and finding meaning (11, 52). For example, Piltch (53) described her recovery process from major depression, in which finding meaningful roles and activities (i.e., parenthood and acquiring a profession), was facilitative not only of recovery, but also had elements that can otherwise be described as PTG. However, as psychiatric symptoms may affect meaning making processes (54–56), we do not know if it affects PTG in individuals who have experienced psychosis. We therefore posed the question of whether PTG is contingent upon meaning making and CSE in people who experienced psychosis.

Trauma, although excruciating, may set in motion a process of rebuilding and positive change (i.e., PTG). However, yet to be thoroughly investigated is the possibility of PTG in individuals who experienced psychosis. To address this issue, we conducted a study to examine the extent to which PTG occurs and to assess the possible mediating effect of CSE and meaning making processes, in such individuals with SMI. We examined the relationships between positive psychotic symptoms, negative symptoms, general psychopathology, and PTG, and hypothesized that:

(a) Positive psychotic symptoms (such as conceptual disorganization and suspicion/persecution), negative symptoms, and general symptoms will be negatively correlated with meaning making processes, CSE, and PTG.
(b) CSE and meaning making processes will be positively correlated with PTG and mediate the relationship between positive psychotic symptoms, negative symptoms, general psychopathological symptoms, and PTG in people who have experienced psychosis.

## Materials and Methods

### Participants

One hundred and twenty-one participants were recruited from community mental health rehabilitation services of the Israeli Ministry of Health. To be eligible for the study, participants had to meet Israeli criteria for having a “psychiatric disability” severe enough to compromise at least 40% of one’s functioning. This is determined by a committee, including a psychiatrist and recognized by National Insurance Institute regulations. The last registry hospital discharge diagnosis of schizophrenia, of whom all Israeli rehab service users are part of, has acceptable sensitivity and specificity assessed against research diagnosis (57); has acceptable stability over time (58); and captured over 90% of cases with schizophrenia disorders based on research criteria in a community sample (59). Inclusion criteria were: men and women over the age of 18, who had been psychiatrically hospitalized in the past, had experienced psychotic symptoms according to their clinicians and corroborated by the Positive and Negative Syndrome Scale (PANSS) [(60) –see measures] clinical interview (administered by the first author) and were eligible to sign an informed consent.

Potential participants were referred to the study by mental health professionals in the rehabilitation services. Participants who wished to take part and met the inclusion criteria contacted the researcher and signed an informed consent form.

Participants completed the questionnaires and were administered the PANSS clinical interview, after which they were paid for their participation. Data were collected by the first author between May 2014 and October 2014. The study protocol was reviewed and approved by the University of Haifa ethics board.

### Measures

#### Demographic Data

Factors such as gender, age, education, and marital status were queried.

#### Clinical History

Diagnosis and time since last hospitalization were determined by psychiatrists and other mental health professionals during the intake procedure.

#### Traumatic History

The Trauma History Screen [THS (61)] is an 18-item instrument used to screen lifetime trauma history. The THS examines high magnitude stressors (HMS), traumatic stressors (TS), and persisting posttraumatic distress, which refers to sudden events that have been found to cause extreme distress. Items include natural disasters, interpersonal traumas, military trauma, etc. For each potentially traumatic event on the scale, participants indicated whether they had ever experienced it in a binary (yes/no) format. The THS has been found to be reliable and have good psychometric properties (61). The THS was translated into Hebrew, back translated, with any language discrepancies resolved by a third bilingual person with trauma expertise. Three psychosis-related items were added, namely: “Have you ever experienced psychosis?” “Have you been psychiatrically hospitalized?” and “Do you suffer from serious mental illness?”

#### Positive, Negative, and General Symptoms

The PANSS (60) is a semi-formalized 30-item interview used to assess affective, motor, cognitive, perceptual, attentional, integrative, and interactive functions in individuals with SMI. The PANSS measures three domains: positive symptoms (7 items; e.g., hallucinations, delusions), negative symptoms (7 items; e.g., blunted affect, social withdrawal), and general symptoms (16 items; e.g., depression, anxiety). The symptoms are assessed on a 7-point scale that represents the severity of psychopathology from 1 (*absent*) to 7 (*extreme*). The summations of ratings range from 7 to 49 for the positive and negative scales and from 16 to 112 for the general symptoms scale. The reliability and validity of the PANSS in Hebrew are well established (62, 63). The internal consistency of the PANSS in the present sample was good (α = 0.86). The PANSS interviews were administered by the first author, an experienced mental health professional, after extensive clinical training on the scale.

#### Posttraumatic Growth Inventory (PTGI)

The PTGI (5) is a 21-item scale that assesses positive changes occurring in response to major adversities. The PTGI is scored on a 6-point Likert scale, from 0 (“*I did not experience this change as a result of my crisis*”) to 5 (“*I experienced this change to a very great extent as a result of my crisis*”). The Hebrew PTGI showed good psychometric properties (64, 65) and was successfully used in previous studies (66). Cronbach’s α for the PTGI factors in the present sample was good to excellent (range α = 0.84–0.92). In the current research, one question was added in which participants were asked to name the traumatic event to which they referred regarding their PTG. PTG summation of ratings range from 0 to 105. A cutoff point regarding PTG level was determined as follow: scores of 45 and below represented none to low PTG levels, whereas scores of 46 and above represented medium to very high PTG levels.

#### Coping Self-Efficacy

The CSE Scale (67) is a 26-item scale that assesses the respondent’s confidence in performing coping behaviors when faced with life challenges and is constructed from three facets: (a) problem-focused coping; (b) ability to stop unpleasant emotions and thoughts, and (c) ability to get support from family and friends. The CSE is scored on a 10-point scale from 0 (“*cannot do it at all*”) to 10 (“*certain I can do it when faced with challenges*”). The CSE has been used in several studies worldwide (68, 69) and has been translated and back translated into Hebrew and English. The internal consistency of the CSE in the present study was excellent (α = 0.93).

#### Meaning in Life

The Meaning in Life Questionnaire [MLQ (51)] is a 10-item scale that was used to identify meaning making processes; namely, the presence of meaning and the search for meaning, on a scale from1 (“*absolutely untrue*”) to 7 (“*absolutely true*”). The MLQ was used in a variety of studies (70, 71) and was translated and back translated into Hebrew and English. Cronbach’s α for the MLQ total score was excellent (α = 0.90).

### Data Analyses

The study hypotheses about mediation were examined regarding (a) PANSS positive, negative, and general symptoms as the independent variable (IV), meaning making processes (MLQ) and CSE as mediators, and post-traumatic growth inventory (PTGI) as the dependent variable (DV). Mediation was examined with Preacher and Hayes’ (72, 73) bootstrapping procedures (MEDIATE, SPSS22.0). To avoid the inflation of alpha due to multiple analyses, the Bonferroni criterion was applied for each table, resulting in a cutoff of *p* = 0.008 (0.05/6 = 0.008). Due to high inter-correlations between the MLQ factors (*r* = 0.59, *p* < 0.001) and between the CSE factors (*r* = 0.62–0.80, *p* < 0.001), total scores of MLQ and CSE factors were used. Due to moderate inter-correlations between the PANSS factors (*r* = 0.53–0.65, *p* < 0.001), the total score was used in the main analyses. For exploratory purposes, in the final part of the study, the three factors were used, each in a separate analysis. Moderate to high inter-correlations were found between the PTGI factors as well (*r* = 0.30–0.67, *p* < 0.001), but because this measure was the DV, each factor was used separately. To understand the nature of marginal relationships found between MLQ and PANSS positive symptoms, the MLQ was split at the median and the PANSS positive symptom factor was split into three equal groups, which were analyzed using a chi-square test.

## Results

### Descriptive Information

Table 1 summarizes the demographic, clinical, and trauma characteristics of the study sample. The participants were an average of 43.8-years old (SD = 11.8). Most of them had schizophrenia or schizoaffective disorder. Over half of the participants (57%, based upon the traumatic history screen) had previous traumatic experiences; two thirds (66.2%) reported events that caused extreme distress, and 47.1% of participants reported exposure to continuous TS. The most common HMS were psychiatric hospitalization (90.1%) and psychotic symptoms (90.9%).

**Table 1:** **Participants’ demographic and clinical characteristics (*N* = 121)**.

Variable	*n*	%
**Gender**		
Male	56	46.3
Female	65	53.7
**Diagnosis**		
Schizophrenia and schizoaffective	95	78.5
Bipolar with past psychotic symptoms	9	7.4
Personality or affective disorders with psychotic symptoms	17	14.1
**Family status**		
Single	70	57.9
Divorced/separated/widowed	32	26.5
Married/living in couple relationship	19	15.6
**Children**		
No children	82	67.8
With children	39	32.2
**Housing status**		
Lives independently	24	19.8
Lives with family	37	30.6
Rehabilitation housing services in the community	60	49.6
**Employment status**		
Not working	33	27.3
Working in community rehabilitation factories	88	72.7
**Psychiatric hospitalization**		
Were psychiatrically hospitalized	119	98.3
No hospitalization	2	1.7
**Time since last psychiatric hospitalization**		
Less than 1 year	15	12.4
1–5 years	48	39.7
More than 5 years	28	23.1
More than 10 years	24	19.8
Did not answer	6	5.0
**Traumatic history (including multiple experiences)**		
Experienced more than five high magnitude stressors	106	87.5
Experienced sexual abuse as a child	31	25.6
Experienced violence as a child	47	39.7
Experienced a sudden death/loss of a loved one	81	66.9

### Correlational Analyses

To assess the relationships between the study variables, Pearson correlations were performed, which are presented in Table 2. Higher PTG was generally related to lower PANSS scores and to higher meaning making and CSE scores. This was found for PTG total scores, as well as for most subscales, except for some of the relationships between PTG and PANSS positive symptoms. In addition, higher PANSS scores were related to lower meaning making and CSE scores, whereas higher meaning making and higher CSE were interrelated. An exception was the marginal correlation between meaning making and PANSS positive symptoms (*p* = 0.076; subsequent lowest *p* value was.081). Correlations between age, years of education, time of hospitalization, and the total PTGI score were not significant, nor were gender differences on any of the measures. Family status had low variance; therefore, analyses were conducted without the control of background variables.

**Table 2:** **Means, SDs, and correlations of the research variables, subscale scores (*N* = 121)**.

Variable, M (SD)	PTGI relation with others	PTGI new possibilities	PTGI personal strength	PTGI spiritual change	PTGI appreciation of life	PANSS total	PANSS positive symptoms	PANSS negative symptoms	PANSS general psychopathology	Meaning total (Meaning in Life Questionnaire)	Coping self-efficacy (CSE) total
PTGI total score 61.16 (21.87)	0.89***	0.86***	0.81***	0.62***	0.78***	−0.38***	−0.20*	−0.38***	−0.36***	0.68***	0.66***
PTGI relation with others 19.91 (*7.98*)		0.67***	0.64***	0.49***	0.61***	−0.41***	−0.26**	−0.42***	−0.34***	0.58***	0.56***
PTGI new possibilities 14.99 (*6.42*)			0.60***	0.48***	0.61***	−0.31***	−0.15	−0.33***	−0.28**	0.61***	0.62***
PTGI personal strength 12.45 (*4.58*)				0.40***	0.65***	−0.28**	−0.16	−0.20*	−0.31***	0.47***	0.54***
PTGI spiritual change 4.86 (*3.41*)					0.30***	−0.21*	−0.05	−0.24**	−0.20*	0.50***	0.35**
PTGI appreciation of life 8.96 (*4.31*)						−0.28**	−0.12	−0.25**	−0.29***	0.55***	0.54***
PANSS total 84.20 (19.33)							0.76***	0.86***	0.91***	−0.39***	−0.40***
PANSS positive symptoms 18.79 (*5.08*)								0.53***	0.57***	−0.16	−0.21*
PANSS negative symptoms 23.73 (*7.61*)									0.65***	−0.34***	−0.31***
PANSS general psychopathology 41.68 (*9.76*)										−0.43***	−0.45***
Meaning total (MLQ) 45.54 (*14.57*)											0.74***
CSE total 139.80 (*50.64*)											

*PTGI, Posttraumatic Growth Inventory; PANSS, Positive and Negative Syndrome Scale*.

*p < 0.05.

**p < 0.01.

***p < 0.001.

To understand the nature of the marginal relationship between meaning making (MLQ) and PANSS positive symptoms, MLQ was split at the median [at score 47 (range 10–70)] and PANSS positive symptoms were split into three equal groups [at scores 16 and 21 (range 9–32)] and analyzed using a chi-square test. Results showed that among those with low positive symptoms, 62% reported high MLQ, whereas among those with high positive symptoms, only 29% reported high MLQ. Among those with moderate positive symptoms, 55% reported high MLQ [χ^2^(2) = 9.17, Cramer’s *V* = 0.275, *p* = 0.010].

To explore which positive symptoms were most strongly related to PTG, we computed the correlation between the severity of positive PANSS items and the MLQ. Conceptual disorganization (PANSS positive, item #2) was negatively correlated to meaning in life (*r*_ρ_ = −0.26, *p* = 0.004) (i.e., higher conceptual disorganization was related to lower MLQ). Moreover, suspicion/persecution (PANSS positive, item #6) was negatively related to MLQ (*r*_ρ_ = −0.18, *p* = 0.05), whereas grandiosity (PANSS positive, item #5) was positively related to MLQ (*r*_ρ_ = 0.18, *p* = 0.04). The other correlations between the individual positive symptoms and MLQ total score were not significant.

### Mediation Analyses

To assess the mediating role of meaning in life (MLQ) and CSE between the PANSS (IV) and PTGI (DV), mediation bootstrapping procedures were used, as mentioned above in the analytical strategy section. As the inter-correlations between the dimensions of PANSS were high (*r* = 0.53–0.65, *p* < 0.001), we first assessed the mediated relationships with the five PTGI dimensions as the outcomes, and the PANSS total score as the IV. That is to say, mediation was examined for (a) PANSS total score, MLQ total score, and PTGI and its dimensions (Table 3), and (b) PANSS total score, CSE total score, and PTGI and its dimensions (Table 4). Due to the theoretical importance of PANSS dimensions, they were examined separately, with MLQ total score and CSE total score. This time, only the PTGI total score was used to avoid redundancy (Table 5).

**Table 3:** **Mediation analyses for Meaning in Life Questionnaire total, Positive and Negative Syndrome Scale total, and Posttraumatic Growth Inventory (total and its five dimensions) scores (*N* = 121)**.

Dependent variable (DV)	Independent variable (IV)	Mediator	IV to mediator	Mediator to DV	Mediation effect	*Z*
			*B* (SE)	*B* (SE)	*B* (SE)	
PTGI total	PANSS total	MLQ total	−0.29*** (0.06)	0.93*** (0.11)	−0.27 (0.07)	4.02***
PTGI relations with others total				0.09** (0.03)	−0.08 (0.02)	3.60***
PTGI new possibilities total				0.25*** (0.03)	−0.07 (0.02)	3.97***
PTGI personal strength total				0.14*** (0.03)	−0.04 (0.01)	3.07**
PTGI spiritual change total				0.12*** (0.02)	−0.03 (0.01)	3.89***
PTGI appreciation of life total				0.15*** (0.02)	−0.05 (0.01)	3.40***

**p < 0.01, ***p < 0.001.

**Table 4:** **Mediation analyses for Positive and Negative Syndrome Scale total, coping self-efficacy total, and Posttraumatic Growth Inventory (total and its five dimensions) scores (*N* = 121)**.

Dependent variable (DV)	Independent variable (IV)	Mediator	IV to mediator	Mediator to DV	Mediation effect	*Z*
			*B* (SE)	*B* (SE)	*B* (SE)	
PTGI total	PANSS total	CSE total	−1.05*** (0.22)	0.26*** (0.03)	−0.27 (0.08)	3.53***
PTGI relations with others total				0.07*** (0.01)	−0.08 (0.02)	3.33***
PTGI new possibilities total				0.07*** (0.01)	−0.08 (0.02)	3.85***
PTGI personal strength total				0.05*** (0.01)	−0.05 (0.02)	2.93**
PTGI spiritual change total				0.02** (0.01)	−0.02 (0.01)	2.88**
PTGI appreciation of life total				0.04*** (0.01)	−0.05 (0.02)	2.83**

**p < 0.01, ***p < 0.001.

**Table 5:** **Mediation analyses for the dimensions of Positive and Negative Syndrome Scale, Meaning in Life Questionnaire total, coping self-efficacy total, and Posttraumatic Growth Inventory total (*N* = 121)**.

Dependent variable (DV)	Independent variable (IV)	Mediator	IV to mediator	Mediator to DV	Mediation effect	*Z*
			*B* (SE)	*B* (SE)	*B* (SE)	
PTGI total	PANSS positive symptoms total	MLQ total	−0.46 (0.26)	0.99*** (0.10)	−0.46 (0.27)	1.71
		CSE total	−2.08 (0.89)	0.28*** (0.03)	−0.58 (0.28)	2.09
	PANSS negative symptoms total	MLQ total	−0.64*** (0.17)	0.93*** (0.11)	−0.60 (0.17)	3.62***
		CSE total	−2.06*** (0.58)	0.26*** (0.03)	−0.53 (0.17)	3.13**
	PANSS general psychopathology total	MLQ total	−0.64*** (0.12)	0.96*** (0.11)	−0.61 (0.13)	4.77***
		CSE total	−2.32*** (0.43)	0.27*** (0.03)	−0.62 (0.15)	4.08***

**p < 0.01, ***p < 0.001.

Results of the role of MLQ regarding the relationship between PANSS total and PTGI total and its dimensions showed that MLQ mediated the relationship between the PANSS total score and the PTGI total score and its dimensions. In other words, less severe symptoms on the PANSS were associated with higher meaning making (MLQ total score), which in turn were related to a higher PTG on the PTGI.

Results regarding the mediating role of CSE total on PANSS total and PTGI total and dimensions showed that more severe symptoms on the PANSS were related to lower CSE on the CSE, which in turn were related to a lower level of PTG.

Finally, results showed that both MLQ and CSE mediated the relationship between PANSS negative symptoms and PANSS general psychopathology, and PTGI (see Table 5). Results also showed that lower scores on PANSS negative symptoms and PANSS general symptoms were related to higher MLQ scores and higher CSE scores, which were then related to higher PTGI total scores. However, mediation was not significant for PANSS positive symptoms and PTGI total score.

## Discussion

In the current study, 80% of participants indicated medium to very high levels of PTG. The mean score on overall PTG was moderately high (M = 63), which is consistent with PTG scores in other populations such as earthquake survivors (74), bereaved parents (75), and women with breast cancer (76). Greater severity across all symptoms (positive, negative, and general psychopathological symptoms) was associated with worse CSE, less meaning making, and less PTG. One possible explanation for these associations may be that distress and distraction, which is associated with the above said symptoms, can thwart coping efforts and the ability to develop meaning from experiences, thus hamper PTG. Further findings, consistent with our hypotheses, indicated that CSE and meaning mediated the adverse impact of negative symptoms and general symptoms on PTG among people with SMI who have experienced psychosis. In addition, screening for traumatic history elicited elevated levels of traumatic exposure and cumulative trauma; 87.5% indicated experiencing more than five HMS during their lives, 25.6% experienced sexual abuse as children, and 39.7% experienced violence as children. These elevated levels of traumatic exposure replicate previous research suggesting high levels of trauma exposure in SMI (9, 77, 78).

As hypothesized, positive symptoms had a significant negative relationship with CSE and PTG. This may be explained by the cognitive impairments that often accompany psychotic symptoms (79), which might be caused by aberrant assignment of salience to external objects and internal representations (54, 80–82). Indeed, reality distortions and conceptual disorganization might hamper cognitive processing (e.g., thoughtful reflection and awareness) and cause higher levels of distress alongside distressing reappraisal processes, which might lead to the development of less effective coping means (50, 83–85). The accompanying distress might also elevate the feelings of uncontrollability and arousal that are inherent to psychotic symptoms (86–88), and further affect the appraisal of coping efficacy, actual coping (42, 89, 90), and possibly PTG. Another possible explanation for the negative relationship between positive symptoms, CSE and PTG, can stem from a neurocognitive perspective regarding deficits in executive function, which lead to passive avoidant coping strategies (89, 91) and can thus hamper both CSE and PTG. Finally, dopaminergic transmission dysfunction in the prefrontal cortex in schizophrenia and its grave effect on cognitive impairments (such as difficulties in working memory and decision making) (80), may also contribute to poor CSE appraisal and difficulties in experiencing PTG.

As hypothesized, positive symptoms had a significant negative relationship with meaning. This is consistent with previous studies reporting maladaptive processes of misplaced meaning and inaccurate interpretation of external and internal stimulus in psychosis (92, 93). Positive symptoms such as reality distortion and conceptual disorganization could hamper normal emotional processes that allow people to find meaning following adversity. Normal emotional processing is composed, in part, of self-regulation of emotional disturbances (e.g., anxiety, depression, etc.). When hampered, difficulties in meaning making might develop and subsequently hinder PTG. As witnessed by the negative correlation found between conceptual disorganization and meaning making, cognitive disorganization (as manifested by confusion, distress, and elevated levels of emotional stress reactivity) might also affect the emotional and cognitive ability to engage in meaning making (54–56). However, one positive symptom was found to have positive relationship with meaning (i.e., grandiosity; PANSS positive, item #5). This raises two possibilities. First, high levels of grandiosity, such as the belief that one has special powers or abilities, or is famous or very accomplished, may be associated with a sense of discovery and relief that incorporates meaning and the experience of PTG, irrespective of the reality of those beliefs, reflecting the possible illusory part of PTG. The illusory part of PTG may thus be conceptualized as a component of PTG that helps people counterbalance their emotional distress in the face of trauma (32). Alternatively, high levels of grandiosity may merely reflect the psychotic symptoms these people still experience and not PTG.

The results of this study offer support for our hypothesis that negative symptoms and general psychopathological symptoms are negatively correlated with meaning, CSE, and PTG. The relationship between negative symptoms and meaning making may be explained by deficits inherent in negative symptoms, such as lack of motivation (28), over estimation of effort required to achieve goals, and the anticipation of negative outcomes (94, 95). These deficits, in turn, might lead to a lack in drive and energy to commence meaning making processes and thus might hamper PTG. Next, consistent with the literature (90, 96) and as mentioned above, higher levels of negative symptoms were linked to lower CSE levels. This might be because individuals with psychotic disorders have difficulty in anticipating pleasure (97, 98). This deficit in anticipatory pleasure might hamper people’s ability to motivate themselves toward beneficial coping actions, thereby hampering CSE. This is in line with studies showing that people with psychosis perceive threats as less controllable, and that higher levels of negative as well as positive symptoms are linked to more dysfunctional coping strategies (86, 99, 100), which might hinder PTG. Additionally, the negative correlations found between general psychopathological symptoms and meaning making, CSE and PTG, are in accordance with studies that found that higher levels of symptoms were related to higher distress levels, which could hinder the ability to appraise coping abilities and find meaning in people who experienced psychosis (90, 101, 102), and therefore impede PTG.

Finally, our findings offer support for the hypothesis that CSE and meaning making mediate the adverse impact of negative symptoms and general symptoms on PTG among people who have experienced psychosis. This is consistent with PTG literature, which suggests that a certain level of distress (which might be caused by both negative as well as general psychopathological symptoms, as mentioned above) is needed to embark on the journey of meaning making and PTG (31). On the other hand, this may not be the case when extreme or persistent levels of distress are evident, as might be seen in our findings regarding positive symptoms, namely that meaning making and CSE did not mediate the adverse impact of positive symptoms on PTG. This might be due to the potentially adverse consequences of positive symptoms on cognitive and emotional processes (3, 36, 82), which could hamper meaning making, CSE, and ultimately PTG. In addition, the lack of mediation effect may be due to differences among participants regarding when psychotic symptoms were experienced (i.e., currently or in the past), thus effecting the ability to experience PTG.

A number of practice and policy-related implications can be drawn from the current study, including the importance of addressing traumatic exposure phenomena in rehabilitation programs for people with SMI who have experienced psychosis. Indeed, there is a lack of knowledge and treatment for individuals with SMI who also suffer from high levels of trauma exposure accompanied with a potential for re-traumatization due to psychosis itself. Considering the high levels of comorbidity found in this population extant, psychiatric interventions are not adequate for the treatment of the traumatic aspects of their illnesses. On the other hand, our findings suggest that even when facing high levels of symptoms individuals with SMI can experience PTG, contingent upon their ability to engage in meaning making processes and positively appraise their coping abilities. Hence, we suggest that greater emphasis should be placed on teaching these individuals more effective coping strategies, along with both broadening the perspective of recovery in mental health rehabilitation to PTG, and developing interventions promoting meaning making.

Although the current study has a number of strengths, including well-established assessment tools and a good sample size, it has some limitations. First, external validity might be limited due to the selection process. Indeed, participation was voluntary and participants were solely from rehabilitation centers in the community, which might both bias and limit the generalizability of our sample. Second, this study was cross-sectional and thus does not allow tracking of PTG development over time. We therefore recommend recruiting a large sample for longitudinal studies in the field of psychosis and PTG, specifically regarding the dynamics of positive psychotic symptoms and PTG. Finally, since the current study is exploratory in nature, the findings provide only preliminary data regarding mediational effects and further study of longitudinal data is needed to confirm it.

## Ethics Statement

The study protocol was reviewed and approved by the University of Haifa ethics board. One hundred and twenty-one participants were recruited from community mental health rehabilitation services of the Israeli Ministry of Health (MOH). To be eligible for the study, participants had to meet Israeli criteria for having a “psychiatric disability” severe enough to compromise at least 40% of one’s functioning. This is determined by a committee, including a psychiatrist, and recognized by National Insurance Institute regulations. The last registry hospital discharge diagnosis of schizophrenia, of whom all Israeli rehab service users are part of, has acceptable sensitivity and specificity assessed against research diagnosis (57); has acceptable stability over time (58); and captured over 90% of cases with schizophrenia disorders based on research criteria in a community sample (59). Inclusion criteria were: men and women over the age of 18, who had been psychiatrically hospitalized in the past, had experienced psychotic symptoms according to their clinicians and corroborated by the PANSS [(60), see measures] clinical interview (administered by the first author), and were eligible to sign an informed consent. Potential participants were referred to the study by mental health professionals in the rehabilitation services. Participants who wished to take part and met the inclusion criteria contacted the researcher, and signed an informed consent form. Participants completed the questionnaires and were administered the PANSS clinical interview, after which they were paid for their participation. Data were collected by the first author between May 2014 and October 2014. The study protocol was reviewed and approved by the University of Haifa ethics board. Additional considerations: persons with severe mental illness.

## Authors Note

This paper is part of the first author’s requirements to attain a Ph.D. degree.

## Author Contributions

YM: head researcher; substantial contributor to the conception and design of the work; responsible for the acquisition, analysis, and interpretation of data; revising the work critically for important intellectual content; approved final version for publication; agreeing to be accountable for all aspects of the work. MG: advisor; substantial contributor to the conception and design of the work; responsible for the analysis and interpretation of data; drafting and revising the work critically for important intellectual content; approved final version for publication; agreeing to be accountable for all aspects of the work. KM: substantial contributor to the conception and design of the work; responsible for the analysis and interpretation of data; revising the work critically for important intellectual content; approved final version for publication; agreeing to be accountable for all aspects of the work. DR: advisor; substantial contributor to the conception and design of the work; responsible for the analysis and interpretation of data; drafting and revising the work critically for important intellectual content; approved final version for publication; agreeing to be accountable for all aspects of the work.

## Conflict of Interest Statement

The authors declare that the research was conducted in the absence of any commercial or financial relationships that could be construed as a potential conflict of interest. The reviewer OM and handling Editor declared their shared affiliation, and the handling Editor states that the process nevertheless met the standards of a fair and objective review.
