# Supplementary material for: Posttraumatic Growth in Psychosis
Source: Front Psychiatry. 2016 Dec 19;7:202. doi: 10.3389/fpsyt.2016.00202 (PMC5165025; doi:10.3389/fpsyt.2016.00202)
Supplement: Supplementary file 1 [file table_1.pdf]

Table 1

Participants' Demographics and Clinical Characteristics (N=121)

Variable	n	%
Gender		
Male	56	46.3
Female	65	53.7
Diagnosis		
Schizophrenia & Schizoaffective	95	78.5
Bi-polar with past psychotic symptoms	9	7.4
Personality or affective disorders with psychotic symptoms	17	14.1
Family status		
Single	70	57.9
Divorced/separated/widowed	32	26.5
Married/living in couple relationship	19	15.6
Children		
No children	82	67.8
With children	39	32.2
Housing status		
Lives independently	24	19.8
Lives with family	37	30.6
Rehabilitation housing services in the community	60	49.6
Employment status		
Not working	33	27.3
Working in community rehabilitation factories	88	72.7
Psychiatric hospitalization		
Were psychiatrically hospitalized	119	98.3
No hospitalization	2	1.7
Time since last psychiatric hospitalization		
Less than a year	15	12.4
A year to five years	48	39.7
More than 5 years	28	23.1
More than 10 years	24	19.8
Did not answer	6	5.0
Traumatic history (including multiple experiences)		
Experienced more than five high magnitude stressors	106	87.5
Experienced sexual abuse as a child	31	25.6
Experienced violence as a child	47	39.7
Experienced a sudden death/loss of a loved one	81	66.9
