# Supplementary material for: Posttraumatic Growth in Psychosis
Source: Front Psychiatry. 2016 Dec 19;7:202. doi: 10.3389/fpsyt.2016.00202 (PMC5165025; doi:10.3389/fpsyt.2016.00202)
Supplement: Supplementary file 2 [file table_2.pdf]

Table 2

Means, standard deviations and correlations of the research variables, subscale scores (N=121)

Variable, M *(SD)*	PTGI-relation with others	PTGI-new possibi-lities	PTGI-personal strength	PTGI-spiritual change	PTGI-appreciation of life	PANSS total	PANSS positive symptoms	PANSS negative symptoms	PANSS general psycho-pathology	Meaning total (MLQ)	Coping self-efficacy (CSE) total
PTGI-total score 61.16 (21.87)	.89***	.86***	.81***	.62***	.78***	-.38***	-.20*	-.38***	-.36***	.68***	.66***
PTGI-relation with others 19.91 *(7.98)*		.67***	.64***	.49***	.61***	-.41***	-.26**	-.42***	-.34***	.58***	.56***
PTGI- new Possibilities 14.99 (*6.42*)			.60***	.48***	.61***	-.31***	-.15	-.33***	-.28**	.61***	.62***
PTGI- personal strength 12.45 (*4.58*)				.40***	.65***	-.28**	-.16	-.20*	-.31***	.47***	.54***
PTGI-spiritual change 4.86 (*3.41*)					.30***	-.21*	-.05	-.24**	-.20*	.50***	.35**
PTGI-appreciation of life 8.96 (*4.31*)						-.28**	-.12	-.25**	-.29***	.55***	.54***
PANSS total 84.20 (19.33)							.76***	.86***	.91***	-.39***	-.40***
PANSS positive symptoms 18.79 (*5.08*)								.53***	.57***	-.16	-.21*
PANSS negative symptoms 23.73 (*7.61*)									.65***	-.34***	-.31***
PANSS General psychopathology 41.68 (*9.76*)										-.43***	-.45***
Meaning total (MLQ) 45.54 (*14.57*)											.74***
Coping self-efficacy (CSE) total 139.80 (*50.64*)											

Note. PTGI: Posttraumatic Growth Inventory; PANSS: Positive and Negative Syndrome Scale

*p<.05, **p<.01, ***p<.001
