# Supplementary material for: Posttraumatic Growth in Psychosis
Source: Front Psychiatry. 2016 Dec 19;7:202. doi: 10.3389/fpsyt.2016.00202 (PMC5165025; doi:10.3389/fpsyt.2016.00202)
Supplement: Supplementary file 3 [file table_3.pdf]

Table 3

Mediation analyses for MLQ total, PANSS total, and PTGI (total and its five dimensions) scores (N=121)

Dependent Variable (DV)	Independent variable (IV)	Mediator	IV to mediator	Mediator to DV	Mediation effect	Z
			B (SE)	B (SE)	B (SE)	
PTGI total	PANSS total	MLQ total	-0.29***	0.93***	-0.27	4.02***
			(0.06)	(0.11)	(0.07)	
PTGI				0.09**	-0.08	3.60***
Relations with others total				(0.03)	(0.02)	
PTGI				0.25***	-0.07	3.97***
New possibilities total				(0.03)	(0.02)	
PTGI				0.14***	-0.04	3.07**
Personal strength total				(0.03)	(0.01)	
PTGI				0.12***	-0.03	3.89***
Spiritual change total				(0.02)	(0.01)	
PTGI				0.15***	-0.05	3.40***
Appreciation of life total				(0.02)	(0.01)	

Note. **p<.01, ***p<.001
