# Supplementary material for: Posttraumatic Growth in Psychosis
Source: Front Psychiatry. 2016 Dec 19;7:202. doi: 10.3389/fpsyt.2016.00202 (PMC5165025; doi:10.3389/fpsyt.2016.00202)
Supplement: Supplementary file 4 [file table_4.pdf]

Table 4

Mediation analyses for PANSS total, CSE total, and PTGI (total and its five dimensions) scores (N=121)

Dependent Variable (DV)	Independent variable (IV)	Mediator	IV to mediator<br>B (SE)	Mediator to DV<br>B (SE)	Mediation effect<br>B (SE)	Z
PTGI total	PANSS total	CSE total	-1.05***<br>(0.22)	0.26***<br>(0.03)	-0.27<br>(0.08)	3.53***
PTGI<br>Relations with others total				.07***<br>(.01)	-0.08<br>(0.02)	3.33***
PTGI<br>New possibilities total				.07***<br>(.01)	-0.08<br>(0.02)	3.85***
PTGI<br>Personal strength total				.05***<br>(.01)	-0.05<br>(0.02)	2.93**
PTGI<br>Spiritual change total				.02**<br>(.01)	-0.02<br>(0.01)	2.88**
PTGI<br>Appreciation of life total				.04***<br>(.01)	-0.05<br>(0.02)	2.83**

Note. **p<.01, ***p<.001
