# Supplementary material for: Posttraumatic Growth in Psychosis
Source: Front Psychiatry. 2016 Dec 19;7:202. doi: 10.3389/fpsyt.2016.00202 (PMC5165025; doi:10.3389/fpsyt.2016.00202)
Supplement: Supplementary file 5 [file table_5.pdf]

Table 5

Mediation analyses for the dimensions of PANSS, MLQ total, CSE total, and PTGI total (N=121)

Dependent Variable (DV)	Independent variable (IV)	Mediator	IV to mediator	Mediator to DV	Mediation effect	Z
			B (SE)	B (SE)	B (SE)	
PTGI total	PANSS	MLQ total	-0.46	0.99***	-0.46	1.71
	Positive symptoms total		(0.26)	(0.10)	(0.27)	
		CSE total	-2.08	0.28***	-0.58	2.09
			(0.89)	(0.03)	(0.28)	
	PANSS	MLQ total	-0.64***	0.93***	-0.60	3.62***
	Negative symptoms total		(.17)	(0.11)	(0.17)	
		CSE total	-2.06***	0.26***	-0.53	3.13**
			(0.58)	(0.03)	(0.17)	
	PANSS	MLQ total	-0.64***	0.96***	-0.61	4.77***
	General psychopathology total		(.12)	(0.11)	(0.13)	
		CSE total	-2.32***	0.27***	-0.62	4.08***
			(0.43)	(0.03)	(0.15)	

Note. **p<.01, ***p<.001
